# The functional safety assessment of cyber-physical system operation process described by Markov chain

**DOI:** 10.1038/s41598-022-11193-w

**Published:** 2022-04-30

**Authors:** Viacheslav Kovtun, Ivan Izonin, Michal Gregus

**Affiliations:** 1 Vinnytsia National Technical University, Vinnytsia, 21000 Ukraine; 2 Lviv Polytechnic National University, Lviv, 79013 Ukraine; 3 Comenius University in Bratislava, 820 05 Bratislava, Slovak Republic

**Keywords:** Engineering, Mathematics and computing

## Abstract

The functional safety assessment is one of the primary tasks both at the design stage and at the stage of operation of critical infrastructure at all levels. The article's main contribution is the information technology of calculating the author's metrics of functional safety for estimating the instance of the model of the cyber-physical system operation. The calculation of metric criteria analytically summarizes the results of expert evaluation of the system in VPR-metrics and the results of statistical processing of information on the system's operation presented in the parametric space Markov model of this process. The advantages of the proposed approach are the following: the need to process orders of magnitude less empirical data to obtain objective estimates of the investigated system; taking into account the configuration scheme and architecture of the security subsystem of the investigated system when calculating the metric; completeness, compactness, and simplicity of interpretation of evaluation results; the ability to assess the achievability of the limit values of the metric criteria based on the model of operation of the investigated system. The paper demonstrates the application of the proposed technology to assess the functional safety of the model of a real cyber-physical system.

## Introduction

Assessing the functional safety of cyber-physical systems is undoubtedly relevant because new vulnerabilities are constantly identified^1–5^. Numerous facts of successful cyber attacks indicate a lack of security of cyber-physical systems of all levels and classes. The reasons for this (if there is a relevant and rationally designed tiered protection subsystem) are the emergence of new vulnerabilities and the negligence of privileged users. In addition, the cause of malfunctions is the failure to consider the specifics of the functioning of sensor networks (Industrial) Internet of Things.

The only adequate response to new vulnerabilities is periodically or continuously updating protection mechanisms. The latter option involves accumulating large data sets and high costs for their storage and analysis. However, this is not a problem regarding the functional security of the critical infrastructure. The Security Information and Event Management (SIEM) and User and Entity Behavior Analytics (UEBA) subsystems are responsible for storing and analyzing the results of the target cyber-physical system^6–11^. In the field of SIEM and UEBA already operates several commercial software products, including ArcSight ESM, QRadar SIEM, Splunk Enterprise Security, Micro Focus Security ArcSight UBA, Securonix UEBA, Splunk User Behavior Analysis. The practical experience of these solutions has revealed their imperfections in the analysis of causal relationships between the facts of failures and malfunctions and operative information about the operation of target systems.

Recognized information sources^12–14^ such as the National Vulnerability Database (NVD), the Common Weakness Enumeration (CWE), the Common Attack Pattern Enumeration and Classification (CAPEC), MITER Att&ck, etc. are providers of benchmarks for known vulnerabilities. In addition to the essence of known vulnerabilities in these databases, there are metrics for ordering them by degree of danger. However, these metrics are reduced to a single indicator, the value of which can be objectively used only as an additional factor in expert analysis of the real cyber-physical system.

Ensuring the functional safety of cyber-physical systems is a complex problem. This thesis allows us to mention several methodologies related to our object of investigation. These are the following methodologies^15–19^:

- the integration of information;
- the security analysis;
- the analysis of security policies;
- the decisions support in the field of protection;
- the automated control of the protection subsystem (including the based of Security Content Automation Protocol ones);
- the correlation analysis of events;
- the definition of security metrics.

Interestingly, apologists for expert methods of functional safety assessment^20,21^ focus their efforts on developing methodologies to support decision-making and metrics in the field of investigation and summarize the results in the form of profile standards, such as ISO/IEC 61508, for example. Apologists of the methodology of automated control of the protection subsystem^15–17,19,22–24^ define the core of such systems in the mathematical apparatus of probability theory and mathematical statistics, graph theory, and Petri nets, fuzzy logic, Markov chains, artificial intelligence and more. At the same time, the results obtained in this direction are of research interest because applying the obtained models and methods requires large amounts of empirical data and computing power.

The Markov process as a mathematical model for studying complex technical and information (cyber-physical) systems is well known^14,22–36^. Visibility, a high level of adequacy of the mathematical model and a deeply worked out mathematical apparatus of Markov processes make it possible to use it in such areas as control of operation processes, queuing systems, the operational reliability of these types of systems and their components. The main advantages of Markov processes are the ability to build predictively controlled models of the behaviour of a cyber-physical system or a group of its components in time based on statistical information or the results of operational observations. Most often, a Markov process is presented as a model with a probabilistic structure, which allows one to determine the probability of a cyber-physical system falling into one of the states of the process for a certain time or time interval.

One of the most effective ways to significantly reduce the cost of maintenance and repair of cyber-physical systems is the choice of the optimal strategy for their operation. When describing the model of the behaviour of a cyber-physical system using the analytical apparatus of the Markov process, it seems possible to link the probabilistic structure of the change in the state of the system with income or expenses that arise when the system passes from one state of the process to another (for example, the transition of a system from an unfunctional state to a functional one is accompanied by the cost of its repair). With this approach, labour costs for its maintenance are used as the main indicator for analyzing a system, and a model based on Markov processes allows us to estimate the total labour costs for maintaining a system for a certain period of operation, as well as to choose a control strategy in which the costs of operating the system under study will be optimal.

In addition, assessing the functional safety of the cyber-physical systems involves machine learning methods^25–27^. This trend is due to the need to automate the process of detecting in the logs with the results of the operation of the target system of features characteristic of known types of vulnerabilities. This task is semantically related to intelligent data analysis. However, the use of smart technologies in the field investigated in this article is risky because the first ones demonstrate high efficiency in processing the content of balanced and statistically representative data sets. Still, the content of real logs is far from these ideals. Also relevant is the question of the difference between qualitative metrics in intelligent data analysis (classification task in the field of pattern recognition theory) and the field of dependability theory.

Let's accumulate the mentioned information by defining the obligatory attributes of scientific investigation. Thus, the *object* of investigation is the operation process of the cyber-physical system. The *subject* of research is the mathematical apparatus of probability theory mathematical statistics, and the theory of Markov chains. This study *aims* to create information technology for assessing functional safety based on the Markov model of cyber-physical system operation. The *main contribution* of this paper are the following:

1. we have described the life cycle of the cyber-physical system in the context of determining its functional safety in the form of compact and informative metrics;
2. we have created the model of cyber-physical system operation using Markov chain, and have considered:
  the situation of lack of the necessary mechanism in the protection subsystem (new vulnerability);
  the situation when the protection subsystem neutralizes the failure caused by a known vulnerability in one cycle (normal operation of the protection subsystem);
  the situation when the protection subsystem neutralizes the failure caused by a known vulnerability in more than one cycle (system in idle);
3. we have formalized the method of calculating the criteria of the created metric for an instance of the cyber-physical system operation model, taking into account information from etalon databases on known vulnerabilities and empirical information on the results of operation of the investigated system.

## Models and methods

### Research statement

Assume that the set of stable states of the investigated cyber-physical system in discrete time is defined as $S = \left\{ {S_{j} ;j = \left\{ {0,i = \overline{1,n} ,\overline{n + 1,2n} } \right\}} \right\}$, where $S_{0}$ is the operational state and $S_{th} = \left\{ {S_{i} ,i \in I = \overline{{\left\{ {1,n} \right\}}} } \right\} \in S$ is the set of intermediate inoperational states of the system response to *i*-th failure $i \in I$. From the $S_{i} \in S_{th}$-th state, the cyber-physical system can either (if the protection mechanisms neutralize the failure) return to the operational state $S_{0}$, or (otherwise) move to the corresponding final inoperational state $S_{2i} \in S_{f} = \left\{ {S_{2i} ,i \in I = \overline{{\left\{ {1,n} \right\}}} } \right\} \in S$ (states of the set $S_{f}$ differ in consequences from the implementation of the corresponding failures).

Suppose that at the initial moment $t = 0$ of the interval of censored observation, the investigated cyber-physical system is in the state $S_{0}$. Then:

1. A cyber-physical system in state $S_{0}$ at a time $t > 0$ can at the time $t + 1$: (a) with probability $q_{i}$ move to state $S_{i} \in S_{th}$ if the *i*-th failure is realized; (b) with probability $q_{0} = 1 - \sum\nolimits_{i = 1}^{n} {q_{i} }$ will remain in state $S_{0}$.
2. A cyber-physical system in state $S_{i} \in S_{th}$ at a time $t > 0$ can at the time $t + 1$: (a) with probability $r_{i}$ move to state $S_{0}$ (protection mechanisms have neutralized the failure); (b) with the probability $d_{i}$ will remain in the state $S_{i}$ (counteraction of protection mechanisms of failure proceeds); (c) with probability $\tilde{r}_{i} = 1 - r_{i} - d_{i}$ move to state $S_{2i} \in S_{f}$ (failure is not neutralized, so the system becomes inoperational).
3. The cyber-physical system in state $S_{2i} \in S_{f}$ at the time $t > 0$ will remain in this state throughout the censored observation interval.

**Figure 1:**
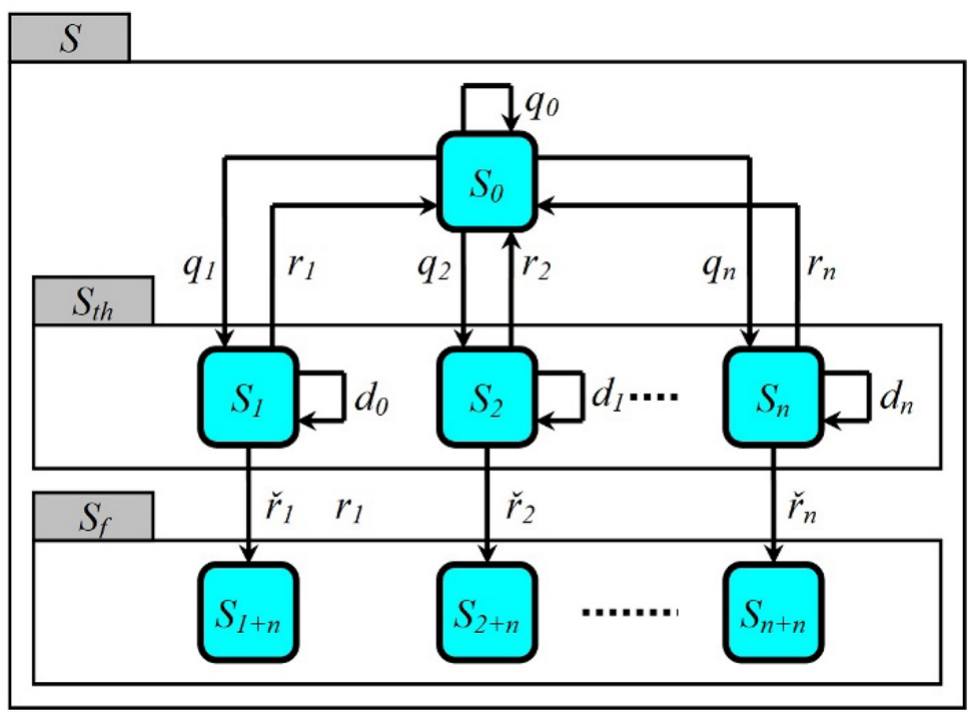
UML state diagrams of the model of the investigated cyber-physical system operation.

These initial provisions indicate that the state of the investigated cyber-physical system at an arbitrary discrete moment of time is recognized only as the state in which it was at the previous moment of time. Thus, the semantic relationship between the states of the set $S$ is determined by the provisions of the theory of Markov chains and can be clearly represented in the form of UML state diagram, visualized in Fig. 1.

The stochastic input parameters of the model of the investigated cyber-physical system operation are organized into such sets with power $n$ as:

- set $Q = \left\{ {q_{i} ;i = \overline{1,n} } \right\}$, which characterizes the probabilities of the corresponding failures;
- set $R = \left\{ {r_{i} ;i = \overline{1,n} } \right\}$, which characterizes the probabilities of neutralization of the respective failures by protection mechanisms for one cycle $\Delta t$;
- the set $D = \left\{ {d_{i} ;i = \overline{1,n} } \right\}$ indicates the probabilities that protection mechanisms' counteraction to the respective failures will last more than one cycle ∆*t*.

The values of the elements of these sets must correspond to the conditions:

1 $$ \left( {\sum\limits_{i = 1}^{n} {q_{i} \le 1} } \right)\& \left( {d_{i} + r_{i} \le 1} \right)\forall i \in I. $$

In order to determine the Markov chain presented in Fig. 1, it is necessary to calculate the probabilities of all $i \in I$ of its states from the set $S$ at time $t$:$\left\{ {p_{i} \left( t \right);i = \overline{0,2n} } \right\} = P\left( t \right)$. The classical formula can describe the process of this calculation in matrix form:

2 $$ P\left( t \right) = P\left( 0 \right)\Pi^{t} . $$

For presented in Fig. 1 of the Markov chain, the matrix of transition probabilities $\Pi$ mentioned in expression (2) is defined as

3 $$ \Pi = \left( {\begin{array}{*{20}c} {q_{0} } & {q_{1} } & {q_{2} } & \ldots & 0 & 0 & 0 & \ldots & 0 \\ {r_{1} } & {d_{1} } & 0 & \ldots & 0 & {\overset{\lower0.5em\hbox{$\smash{\scriptscriptstyle\smile}$}}{r}_{1} } & 0 & \ldots & 0 \\ {r_{2} } & 0 & {d_{2} } & \ldots & 0 & 0 & {\overset{\lower0.5em\hbox{$\smash{\scriptscriptstyle\smile}$}}{r}_{2} } & \ldots & 0 \\ \ldots & \ldots & \ldots & \ldots & \ldots & \ldots & \ldots & \ldots & \ldots \\ {r_{n} } & 0 & 0 & \ldots & {d_{n} } & 0 & 0 & \ldots & {\overset{\lower0.5em\hbox{$\smash{\scriptscriptstyle\smile}$}}{r}_{n} } \\ 0 & 0 & 0 & \ldots & 0 & 1 & 0 & \ldots & 0 \\ 0 & 0 & 0 & \ldots & 0 & 0 & 1 & \ldots & 0 \\ \ldots & \ldots & \ldots & \ldots & \ldots & \ldots & \ldots & \ldots & \ldots \\ 0 & 0 & 0 & \ldots & 0 & 0 & 0 & \ldots & 1 \\ \end{array} } \right). $$

For real cyber-physical systems characterized by large values of $n$ and $t$, the need to raise the matrix (3) to the power of $t$ makes calculating the required values of the set $P\left( t \right)$ according to expression (2) computationally inefficient. But there are two exceptions:

1. If the protection mechanisms of the cyber-physical system do not function, i.e. $R = \left\{ {r_{i} = 0;i = \overline{1,n} } \right\}$. In this case, the elements of the set $P$ can be defined as
  4 $$ p_{0} \left( t \right) = q_{0}^{t} ,t \ge 0, $$
  5 $$ p_{i} \left( t \right) = q_{i} \left( {\frac{{q_{0}^{t} - d_{i}^{t} }}{{q_{0} - d_{i} }}} \right),\quad i = \overline{1,n} ,\,\,t \ge 0, $$
  6 $$ p_{i + n} \left( t \right) = q_{i} \left( {\frac{{\left( {1 - q_{0} } \right)\left( {d_{i}^{t} - 1} \right) - \left( {q_{0}^{t} - 1} \right)\left( {1 - d_{i} } \right)}}{{\left( {q_{0} - d_{i} } \right)\left( {1 - q_{0} } \right)}}} \right). $$
2. If the protection mechanisms of the cyber-physical system have managed to neutralize the failure in one cycle $\Delta t$: $D = \left\{ {d_{i} = 0;i = \overline{1,n} } \right\}$. In this case, the elements of the set $P$ can be defined as:
  7 $$ p_{0} \left( t \right) = \frac{{\gamma_{ + }^{t + 1} - \gamma_{ - }^{t + 1} }}{w},\quad t \ge 0, $$
  8 $$ p_{i} \left( t \right) = \frac{{q_{i} \left( {\gamma_{ + }^{t} - \gamma_{ - }^{t} } \right)}}{w},\quad i = \overline{1,n} ,\,\,t \ge 0, $$
  9 $$ p_{i + n} \left( t \right) = \frac{{q_{i} \left( {1 - r_{i} } \right)}}{w}\left( {\frac{{1 - \gamma_{ + }^{t} }}{{1 - \gamma_{ + } }} - \frac{{1 - \gamma_{ - }^{t} }}{{1 - \gamma_{ - } }}} \right), $$
  where: $w = \sqrt {q_{0} + 4\sum\nolimits_{i = 1}^{n} {q_{i} r_{i} } }$ is a controlled parameter that characterizes the generalized efficiency of the reaction of protection mechanisms and $\gamma_{ \pm } = \frac{{q_{0} \pm w}}{2}$.

For $r_{i} \to 0$ and $d_{i} \to 0$, expressions (4), (5), (6) and (7), (8), (9) coincide in pairs. For both described exceptions, the marginal relationship

10 $$ \mathop {\lim }\limits_{t \to \infty } p_{0} \left( t \right) = \mathop {\lim }\limits_{t \to \infty } p_{1} \left( t \right) = \ldots = \mathop {\lim }\limits_{t \to \infty } p_{n} \left( t \right) = 0, $$

holds, i.e., with a sufficiently large value of $t$, the values of the probabilities of the investigated system in the states of the combined set $S_{0} \cup S_{th}$ are extremely small.

The corresponding boundary relations for the states defined by expressions (6) and (9) from the set $S_{f}$ are formalized as follows:

$$ \mathop {\lim }\limits_{t \to \infty } p_{i + n} \left( t \right) = \frac{{q_{i} }}{{1 - q_{0} }},\quad i = \overline{1,n} , $$

$$ \mathop {\lim }\limits_{t \to \infty } p_{i + n} \left( t \right) = \frac{{q_{i} \left( {1 - r_{i} } \right)}}{{\left( {1 - \gamma_{ + } } \right)\left( {1 - \gamma_{ - } } \right)}},\quad i = \overline{1,n} . $$

It is seen that the limit values of the probabilities of realization of states from the set $S_{f}$ are determined by the values of the initial parameters of the investigated system operation model, i.e., laid at the stage of its design.

### Functional safety assessment technology based on the model of cyber-physical system operation

To determine the claimed technology, it is necessary to formalize the qualitative metrics and the concept of its calculation for an arbitrary instance of the model of cyber-physical system operation in the parametric space of the corresponding attribute of dependability, i.e. functional safety.

According to the material presented in “Research statement”, it can be stated that the set of states of the model of the investigated cyber-physical systems operation $S$ is a conglomerate of sets of states $S_{0}$, $S_{th}$, $S_{f}$: $S = S_{0} \cup S_{th} \cup S_{f}$, the probabilities of realization of which elements are formalized in the transition matrix (3). If we analyze the conglomerate of sets $S = S_{0} \cup S_{th} \cup S_{f}$ from the standpoint of the structure shown in Fig. 1 of the graph, it can be stated that the states $S_{i} \in S_{0} \cup S_{th}$, $i = \overline{0,n}$, are transient and the states $S_{i + n} \in S_{f}$, $i = \overline{1,n}$ are finite.

We define in the matrix of transient probabilities (3) fragments, which, respectively, characterize the transient and final states of the model of the investigated system operation:

11 $$ Q\left( \Pi \right) = \left( {\begin{array}{*{20}c} {q_{0} } & {q_{1} } & {q_{2} } & \ldots & 0 \\ {r_{1} } & {d_{1} } & 0 & \ldots & 0 \\ {r_{2} } & 0 & {d_{2} } & \ldots & 0 \\ \ldots & \ldots & \ldots & \ldots & \ldots \\ {r_{n} } & 0 & 0 & \ldots & {d_{n} } \\ \end{array} } \right), $$

12 $$ R\left( \Pi \right) = \left( {\begin{array}{*{20}c} 0 & 0 & \ldots & 0 \\ {\overset{\lower0.5em\hbox{$\smash{\scriptscriptstyle\smile}$}}{r}_{1} } & 0 & \ldots & 0 \\ 0 & {\overset{\lower0.5em\hbox{$\smash{\scriptscriptstyle\smile}$}}{r}_{2} } & \ldots & 0 \\ 0 & 0 & \ldots & {\overset{\lower0.5em\hbox{$\smash{\scriptscriptstyle\smile}$}}{r}_{n} } \\ \end{array} } \right). $$

Considering the content of matrices (11) and (12), we present a matrix of transition probabilities $\Pi$ in block form:

$$ \Pi = \left( {\begin{array}{*{20}c} {Q\left( \Pi \right)} & {R\left( \Pi \right)} \\ 0 & {I\left( \Pi \right)} \\ \end{array} } \right), $$

where $I\left( \Pi \right)$ is a unit matrix of dimension $n \times n$.

Considering the limit relations (10), for $t \to \infty$ we write: $Q^{t} \left( \Pi \right) \to 0$, where it is obvious that the absolute values of the eigenvalues of the matrix $Q\left( \Pi \right)$ are strictly less than one. This, in turn, means that the inverse form of the nondegenerate matrix $\left( {I\left( \Pi \right) - Q\left( \Pi \right)} \right)$ can be represented as follows:

13 $$ A = \left( {Q\left( \Pi \right) - I\left( \Pi \right)} \right)^{ - 1} = I\left( \Pi \right) + Q\left( \Pi \right) + Q^{2} \left( \Pi \right) + \cdots $$

The element's value $a_{ij} \in A$ shows how many times the studied Markov process from the state $S_{j}$ will reach the state $S_{i}$. We interpret this definition in the context of the task of finding the metrics of functional safety assessment based on the model of the cyber-physical system operation.

Let the process of the investigated cyber-physical system operation start from the state $S_{0}$. Then we estimate the time of the inoperation of this process to consider the stochastic value of the parameter $T$, which is equal to the number of transitions between states from the combined set $S_{0} \cup S_{th}$ until the process enters one of the states from the set $S_{f}$. The mathematical expectation of the stochastic parameter $T$ is determined by interpreting the contents of the matrix (13): $\tau = \sum\nolimits_{j = 1}^{n + 1} {a_{1j} }$.

In the transition from the block representation of the matrix $A$ to the form (11), the newly obtained expression can be redefined as follows:

14 $$ \tau = \frac{{\sum\nolimits_{i = 1}^{n} {q_{i} \prod\nolimits_{j = 1}^{n} {\left( {1 - d_{j} \left( {1 - \delta_{ij} } \right)} \right)} } + \prod\nolimits_{j = 1}^{n} {\left( {1 - d_{j} } \right)} }}{{\sum\nolimits_{i = 1}^{n} {q_{i} \prod\nolimits_{j = 1}^{n} {\left( {1 - d_{j} - r_{j} \delta_{ij} } \right)} } }}, $$

where $\delta_{ij}$ is the corresponding Kronecker delta.

If the set of potential failures is a priori defined in the form $Q$, then the parameter $\tau$ can be expressed as some function in the form $\tau = f\left( {Q,R,D} \right)$, continuous in the domain of its arguments. The range of valid values of the parameter $\tau$ is defined as $\left[ {\tau_{\min } ,\infty } \right)$, where $\tau = f\left( {Q,0,0} \right)$ or $\tau_{\min } = 1 + \left( {\sum\nolimits_{i = 1}^{n} {q_{i} } } \right)^{ - 1}$.

As noted earlier, the probability of inoperation of the investigated cyber-physical system due to the implementation of the *i*-th failure despite the opposition of protective mechanisms is equal to $$\overset{\lower0.5em\hbox{$\smash{\scriptscriptstyle\smile}$}}{r}_{i}$$. Let us estimate the losses from realizing such an event by a positive discrete value $$u_{i} \in U = \left\{ {u_{i} ;i = \overline{1,n} } \right\}$$. Let us summarize these values as the corresponding risk factor $$f_{i} = \overset{\lower0.5em\hbox{$\smash{\scriptscriptstyle\smile}$}}{r}_{i} u_{i}$$. The mathematical expectation of such a stochastic quantity as a risk factor is defined as $$\varphi = \sum\nolimits_{i = 1}^{n} {\overset{\lower0.5em\hbox{$\smash{\scriptscriptstyle\smile}$}}{r}_{i} u_{i} }$$.

We formalize the expression for calculating the parameter $\varphi$ in terms of the Markov chain visualized in Fig. 1. Let's raise the matrix of transition probabilities $\Pi$ presented in block form to the power $t$:

15 $$ \Pi^{t} = \left( {\begin{array}{*{20}c} {Q^{t} \left( \Pi \right)} & {\varphi \left( {\sum\limits_{k = 0}^{t - 1} {Q^{k} \Pi } } \right)} \\ 0 & {I\left( \Pi \right)} \\ \end{array} } \right). $$

The absolute values of the eigenvalues of the matrix $Q\left( \Pi \right)$ are strictly smaller than unity, so for $t \to \infty$ the following boundary relations are satisfied: $Q^{t} \left( \Pi \right) \to 0$, $\sum\nolimits_{k = 0}^{t} {Q^{k} \left( \Pi \right)} \to A$, where we have already mentioned the matrix in expression (13). We define the form of the matrix (15) for $t \to \infty$:

$$ \mathop {\lim }\limits_{t \to \infty } \Pi^{t} = \left( {\begin{array}{*{20}c} 0 & {\varphi A} \\ 0 & {I\left( \Pi \right)} \\ \end{array} } \right). $$

Let the Markov model of the investigated system at time $t = 0$ be in the state $S_{0}$, then, at $t \to \infty$, we write:

16 $$ \overset{\lower0.5em\hbox{$\smash{\scriptscriptstyle\smile}$}}{r}_{i} = \mathop {\lim }\limits_{t \to \infty } p_{i + n} \left( t \right) = \frac{{q_{i} \prod\nolimits_{j = 1}^{n} {\left( {1 - d_{j} - r_{j} \delta_{ij} } \right)} }}{{\sum\nolimits_{k = 1}^{n} {q_{k} } \prod\nolimits_{j = 1}^{n} {\left( {1 - d_{j} - r_{j} \delta_{ij} } \right)} }},\quad i = \overline{1,n} . $$

Based on expression (16) for the parameter $\varphi$ we write:

17 $$ \varphi = \frac{{\sum\nolimits_{i = 1}^{n} {q_{i} u_{i} } \prod\nolimits_{j = 1}^{n} {\left( {1 - d_{j} - r_{j} \delta_{ij} } \right)} }}{{\sum\nolimits_{k = 1}^{n} {q_{k} } \prod\nolimits_{j = 1}^{n} {\left( {1 - d_{j} - r_{j} \delta_{ij} } \right)} }}. $$

Expressions (14) and (17) allow calculating the value of the required metric $\left\{ {\tau ,\varphi } \right\}$ ($\tau$ is the mathematical expectation of the time till the cyber-physical system inoperation, $\varphi$ is the mathematical expectation of risk factor) for an instance of the Markov model of the operation of investigated cyber-physical system characterized by the content of the sets conglomerate $\left\{ {Q,D,R,U} \right\}$. Also, an important parameter is the positive integer value of the duration of the cycle $\Delta t$, which means the minimum time interval after which the investigated system can change its state.

In general, the number of fixed parameters for calculating the metric $\left\{ {\tau ,\varphi } \right\}$ is equal to $4n + 1$, where $n$ is the number of potential categorized failures, which in modern cyberspace exceeds $1.5 \times 10^{5}$. However, this impressive number is an absolute one. More specifically, current failures for cyber-physical systems are justified and ranked according to the degree of danger in such open vulnerability assessment systems^14,28,29^ as Damage, Reproducibility, Exploitability, Affected users, Discoverability (DREAD); Common Vulnerability Scoring System (CVSS); Vulnerability Priority Rating (VPR).

In the future, the authors will focus on the *VPR* system. Here is analytical information in favour of this choice. Not only publicly available technical data but also cyber intelligence is used to address vulnerabilities in the VPR system. Empirical studies^28,29^ have shown that the upgrade of the information and communication system to address 400 critical vulnerabilities detected by VPR has shown the same effect on increasing functional safety as the upgrade of the base version of the same system to address 9000 critical vulnerabilities, detected using the CVSSv3 system. This result convincingly proves that the catalogue of vulnerabilities in the VPR system is organized more rationally than analogues.

Let the $M = \overline{1,m}$ vulnerabilities be identified in the investigated cyber-physical system at the pre-release testing stage^30–32^. Let $v_{\alpha ,i}$ be the value of the base VPR metric for vulnerability $\alpha$, which can lead to failure $i$ (this cause-and-effect relationship we identify as $V_{\alpha ,i}$), where $\alpha = \overline{{1,m_{i} }}$, $i = \overline{1,n}$, $\sum\nolimits_{i = 1}^{n} {m_{i} } = m$ (each vulnerability $\alpha$ can lead to only one failure *i*). It is possible to predict the existence of a certain functional relationship between the probability of *i*-th failure $q_{i}$ and the value of the VPR-metric $v_{\alpha ,i}$. Naturally, the more vulnerabilities that can cause the *i*-th failure, the greater the probability $q_{i}$ against the background of analogues will be (remember that $\sum\nolimits_{i = 1}^{n} {q_{i} } = 1$). In the first approximation, we formalize this functional dependence as follows:

18 $$ q_{i} = \alpha k_{i} ,\quad i = \overline{1,n} , $$

where $\alpha$ is the positive weighting coefficient, and the parameter $k_{i}$ is calculated by the expression

19 $$ k_{i} = \frac{{\sum\nolimits_{\alpha = 1}^{{m_{i} }} {v_{\alpha ,i} } }}{{\sum\nolimits_{j = 1}^{n} {\sum\nolimits_{l = 1}^{{m_{j} }} {v_{jl} } } }}. $$

Analysis of expression (19) allows us to state that the parameter $k_{i}$ and available in the presented in Fig. 1 Markov chain^33,34^, the probability of neutralization of the *i*-th failure $\left( {1 - d_{i} } \right)^{ - 1} r_{i}$ are also functionally related, which in the first approximation is described by the expression:

20 $$r_{i} = \beta \left( {1 - d_{i} } \right)k_{i} ,\quad i = \overline{1,n}$$

where $\beta$ is a positive weighting coefficient.

We connect the author's metric $\left\{ {\tau ,\varphi } \right\}$ with the VPR metric by summarizing expressions (14) and (18) and (17) and (20). In accordance:

21 $$ \tau = {{\left( {\frac{1}{\alpha } + \sum\limits_{i = 1}^{n} {\frac{{k_{i} }}{{1 - d_{i} }}} } \right)} \mathord{\left/ {\vphantom {{\left( {\frac{1}{\alpha } + \sum\limits_{i = 1}^{n} {\frac{{k_{i} }}{{1 - d_{i} }}} } \right)} {\left( {1 - \beta \sum\limits_{i = 1}^{n} {k_{i}^{2} } } \right)}}} \right. \kern-\nulldelimiterspace} {\left( {1 - \beta \sum\limits_{i = 1}^{n} {k_{i}^{2} } } \right)}}, $$

22 $$ \varphi = {{\left( {\sum\limits_{i = 1}^{n} {u_{i} k_{i} \left( {1 - \beta k_{i} } \right)} } \right)} \mathord{\left/ {\vphantom {{\left( {\sum\limits_{i = 1}^{n} {u_{i} k_{i} \left( {1 - \beta k_{i} } \right)} } \right)} {\left( {1 - \beta \sum\limits_{i = 1}^{n} {k_{i}^{2} } } \right)}}} \right. \kern-\nulldelimiterspace} {\left( {1 - \beta \sum\limits_{i = 1}^{n} {k_{i}^{2} } } \right)}}. $$

Expressions (21), (22) are formulated taking into account the rationing $\sum\nolimits_{i = 1}^{n} {k_{i} } = 1$. Note that in the expression (22) for the calculation of the mathematical expectation of the risk factor $\varphi$, the parameters $\alpha$ and $d_{i} \in D$ are absent.

The values of weighting coefficients $\alpha$, $\beta$ and parameters $d_{i} \in D$ are proposed to be determined by expert evaluation (at the design stage of the investigated cyber-physical system), or as a result of statistical analysis of the results of the censored period of its operation (for already accepted into operation cyber-physical system). Let's explore the latter option in more detail.

Let the content of the logs of the investigated cyber-physical system be sufficiently statically representative to calculate:

- the mathematical expectation of the number of cycles between failures $\left\langle {T^{ * } } \right\rangle$;
- the mathematical expectation of the number of cycles required by the protective mechanisms to neutralize the *i*-th failure $\left\langle {d_{i}^{ * } } \right\rangle$;
- share of successfully neutralized failures $\left( {p_{r}^{ * } } \right)$.

In terms of presented in Fig. 1 the Markov chain, the estimation of these parameters can be indirectly calculated by the relevant expressions:

$$ \left\langle {T^{ * } } \right\rangle_{ \approx } = \left( {\sum\limits_{i = 1}^{n} {q_{i} } } \right)^{ - 1} ,\,\left\langle {d_{i}^{ * } } \right\rangle_{ \approx } = \left( {1 - d_{i} } \right)^{ - 1} , $$

23 $$ \left( {p_{r}^{ * } } \right)_{ \approx } = {{\sum\limits_{i = 1}^{n} {\left( {{{q_{i} r_{i} } \mathord{\left/ {\vphantom {{q_{i} r_{i} } {\left( {1 - d_{i} } \right)}}} \right. \kern-\nulldelimiterspace} {\left( {1 - d_{i} } \right)}}} \right)} } \mathord{\left/ {\vphantom {{\sum\limits_{i = 1}^{n} {\left( {{{q_{i} r_{i} } \mathord{\left/ {\vphantom {{q_{i} r_{i} } {\left( {1 - d_{i} } \right)}}} \right. \kern-\nulldelimiterspace} {\left( {1 - d_{i} } \right)}}} \right)} } {\sum\limits_{i = 1}^{n} {q_{i} } }}} \right. \kern-\nulldelimiterspace} {\sum\limits_{i = 1}^{n} {q_{i} } }}. $$

Substituting expressions (23) into expressions (18), (20), we determine the estimates for the weighting coefficients $\alpha$, *β* and parameters $d_{i} \in D$:

$$ \alpha_{ \approx } = {1 \mathord{\left/ {\vphantom {1 {\left\langle {T^{ * } } \right\rangle_{ \approx } }}} \right. \kern-\nulldelimiterspace} {\left\langle {T^{ * } } \right\rangle_{ \approx } }},\,\beta_{ \approx } = {{\left( {p_{r}^{ * } } \right)} \mathord{\left/ {\vphantom {{\left( {p_{r}^{ * } } \right)} {\sum\limits_{i = 1}^{n} {k_{i}^{2} } }}} \right. \kern-\nulldelimiterspace} {\sum\limits_{i = 1}^{n} {k_{i}^{2} } }},\,\left( {d_{i} } \right)_{ \approx } = 1 - \frac{1}{{\left\langle {d_{i}^{ * } } \right\rangle }}, $$

based on which we analytically express the estimates for the metric $\left\{ {\tau ,\varphi } \right\}$:

24 $$ \tau_{ \approx } = \frac{{\left\langle {T^{*} } \right\rangle_{ \approx } + \sum\nolimits_{i = 1}^{n} {\left\langle {d_{i}^{*} } \right\rangle_{ \approx } k_{i} } }}{{1 - \left( {p_{r}^{*} } \right)_{ \approx } }}, $$

25 $$ \varphi_{ \approx } = \frac{1}{{1 - \left( {p_{r}^{*} } \right)_{ \approx } }}\left( {\sum\limits_{i = 1}^{n} {u_{i} k_{i} } - \left( {p_{r}^{*} } \right)_{ \approx } \frac{{\sum\nolimits_{i = 1}^{n} {u_{i} k_{i}^{2} } }}{{\sum\nolimits_{i = 1}^{n} {k_{i}^{2} } }}} \right). $$

If inequality (26) holds, then expressions (25), (27) can be used to calculate the metric $\left\{ {\tau ,\varphi } \right\}$. Constraint

26 $$ \left( {p_{r}^{ * } } \right)_{ \approx } \le \mathop {\min }\limits_{i} \left\{ {{{\sum\limits_{j = 1}^{n} {k_{j}^{2} } } \mathord{\left/ {\vphantom {{\sum\limits_{j = 1}^{n} {k_{j}^{2} } } {k_{i} }}} \right. \kern-\nulldelimiterspace} {k_{i} }}} \right\} $$

is formulated due to the extension to the parameters calculated by expression (23) the condition (1).

Finally, the values of the parameters $u_{i} \in U$, which characterize the losses associated with the inability of the protective mechanisms of the investigated instance (class) of cyber-physical systems to neutralize the *i*-th failure, should be assessed purely by an expert method^35–37^.

## Results

As an example, we use the technology presented in “Models and methods” to assess the functional safety of the model of a real cyber-physical system at the Situation Center of the Department of Information Technology (DIT) of Vinnytsia City Council (VCC) (Vinnytsia, Ukraine). This information and communication system was taken into operation in 2018 and is constantly evolving to improve the implemented services and add new ones. Currently, this information and communication system manages traffic lights on the roads of Vinnytsia. It supports the uninterrupted operation of the data center, which stores video streams from more than 1 k video cameras located in the city.

Collected of confidential information in the system is open only to authorized employees of the Vinnytsia City Council, the National Police of Ukraine, the Security Service of Ukraine, etc. In order for these privileged persons to have prompt access to the relevant information, a local network was created consisting of data center servers, communication equipment, workstations, and software. In normal operation, this LAN is not isolated from the WWW. However, the processing, storage, and audit of confidential information are carried out by a specialized relational database management system, access to which is organized through a specialized web interface. Data, databases and management system, web interface—all these components are located on dedicated servers.

We imitate a situation where attackers exert a deliberate influence on the information and communication system of the Situation Center, which threatens the functional safety of the latter. Attackers seek information about network architecture, workstations, servers, operating systems, user accounts and more. Analysis of this information can identify hardware and software vulnerabilities, some of which may not fall within the scope of the protection subsystem.

In the realities of modern cyberspace, exploits are often created based on data collected as a result of:

- $In_{1}$ (Apache): analysis of internal and outgoing network traffic, the mechanism for supporting remote access;
- $$In_{2}$$: buffer overflow;
- $$In_{3}$$: SQL injection.

The analysis of the logs of the information and communication system of the Situation Center revealed the following categorized vulnerabilities^38–40^:

- $In_{1}$: (a) CVE-2019-9511, (b) CVE-2015-5206, (c) CVE-2019-9512, (d) CVE-2020-9481, (e) CVE-2020-17509;
- $In_{2}$: (a) CVE-2008-0127, (b) CVE-2007-6593, (c) CVE-2021-36301, (d) CVE-2019-18805, (e) CVE-2017–6745;
- $In_{3}$: (a) CVE-2021-45253, (b) CVE-2022-22055, (c) CVE-2021-45814, (e) CVE-2021-44599, (e) CVE-2020-0060.

**Figure 2:**
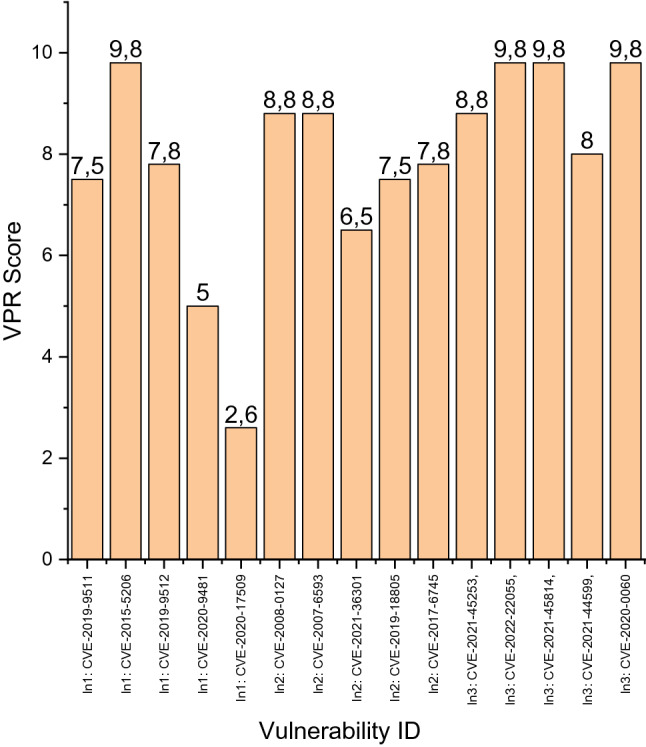
Values of VPR metrics for vulnerabilities mentioned in sets $In_{1}$–$In_{3}$.

A full description of these vulnerabilities can be found at https://www.cvedetails.com/. Note that at the request of VCC administration, the sets $In_{1}$–$In_{3}$ do not contain a complete list of vulnerabilities identified in the investigated system. However, these data are sufficient to demonstrate the functionality of the technology presented in “Models and methods”. The values of the VPR metric for the vulnerabilities listed in the sets $In_{1}$–$In_{3}$ are clearly presented in Fig. 2.

Using the presented in Fig. 2 data $v_{i,\alpha }$, $i = \overline{1,3}$, $\alpha = \overline{1,5}$, by expression (19) we calculate the values of the coefficients $k_{i}$: $K = \left\{ {0.3331,0.3910,0.2764} \right\}$. For further calculations, it is necessary to determine the duration of the cycle $\Delta t$. Analysis of the logs of the investigated system allows us to establish it as equal to the day: 24 [h]. Based on the known description of the identified vulnerabilities, the involved experts estimated the average duration of the impact of each *i*-th type of vulnerabilities, $i = \overline{1,3}$, on the investigated system as follows: $\left\langle {D^{ * } } \right\rangle_{ \approx } = \left\{ {96,24,60} \right\}$ [h], and the estimated loss $u_{i}$ from their implementation as follows: $U = \left\{ {0.2,0.2,0.6} \right\}$ [c.u.] We substitute the obtained values into expressions (24), (25) and get object-oriented expressions for estimating the criteria of the author's metric $\left\{ {\tau ,\varphi } \right\}$:

27 $$ \tau_{ \approx } = \frac{{2.4151 + \left\langle {T^{ * } } \right\rangle_{ \approx } }}{{1 - \left( {p_{r}^{ * } } \right)_{ \approx } }}, $$

28 $$ \varphi_{ \approx } = \frac{{0.3562 - 0.3795\left( {p_{r}^{ * } } \right)_{ \approx } }}{{1 - \left( {p_{r}^{ * } } \right)_{ \approx } }}. $$

Now we define the object-oriented condition (26) for the application of estimates (27): $\left( {p_{r}^{ * } } \right)_{ \approx } < \max \left( {p_{r}^{ * } } \right) = 0.8702$.

**Figure 3:**
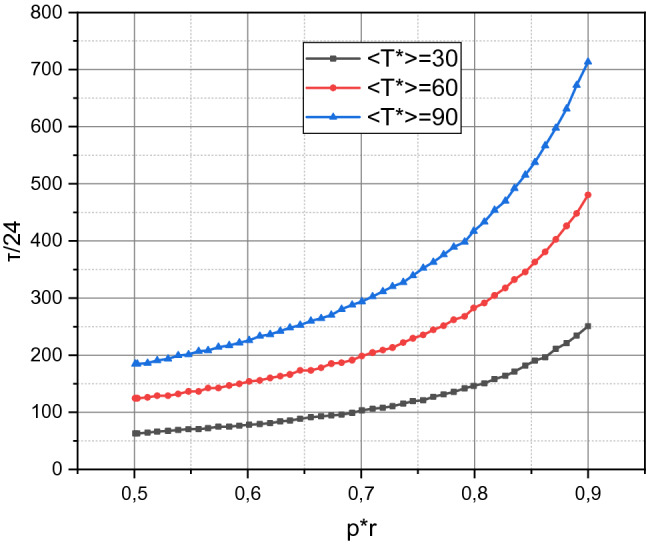
Calculated dependences $$\tau_{ \approx } = f\left( {\left( {p_{r}^{ * } } \right)_{ \approx } ,\left\langle {T^{{}} } \right\rangle_{ \approx } = \left\{ {30,40,60} \right\}} \right)$$.

By changing the parameter's value $\left( {p_{r}^{ * } } \right)_{ \approx }$ at a fixed value of the parameter $\left\langle {T^{ * } } \right\rangle_{ \approx } = const$, we calculate the dependence of $\tau_{ \approx } = f\left( {\left( {p_{r}^{ * } } \right)_{ \approx } ,\left\langle {T^{ * } } \right\rangle_{ \approx } } \right)$ for the investigated system using expression (27) and present the results in Fig. 3.

The dependence $\tau_{ \approx } = f\left( {\left( {p_{r}^{ * } } \right)_{ \approx } ,\left\langle {T^{ * } } \right\rangle_{ \approx } } \right)$ is chosen not by chance because it has an application, which is to determine the minimum threshold value $\left( {p_{r}^{ * } } \right)_{ \approx }$, at which the value of the criterion $\tau$ will not be less than the specified value $\tau_{0}$. For the investigated information and communication system, this definition is embodied in the expression

29 $$ \left( {p_{r}^{ * } } \right) \ge 1 - \left( {2.4152 + \left\langle {T^{ * } } \right\rangle_{ \approx } } \right)\tau_{0}^{ - 1} . $$

**Figure 4:**
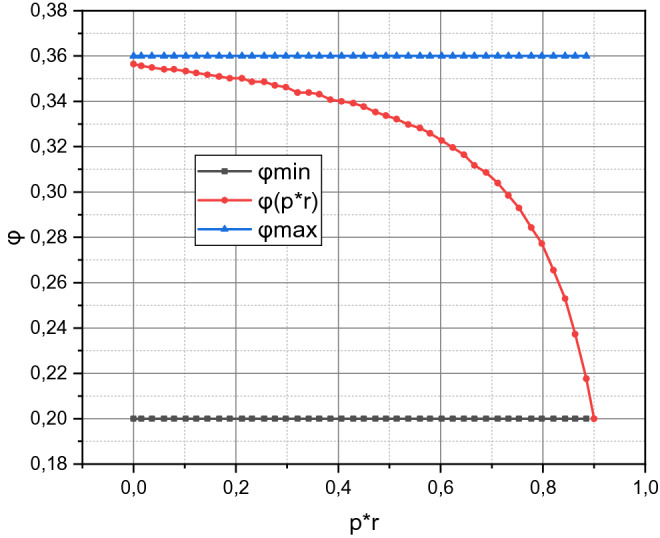
The calculated dependence $\varphi_{ \approx } = f\left( {\left( {p_{r}^{ * } } \right)_{ \approx } } \right)$ in the confidence interval $\left[ {\varphi_{\min } = 0.2,\varphi_{\max } = 0.36} \right]$.

By changing the parameter's value $\left( {p_{r}^{ * } } \right)_{ \approx }$, we calculate the dependence of $\varphi_{ \approx } = f\left( {\left( {p_{r}^{ * } } \right)_{ \approx } } \right)$ for the investigated system using expressions (28) and present the results in Fig. 4.

As we noted in the formalization of expression (17), the change in the parameter $\left\langle T \right\rangle$ does not affect the value of the criterion $\varphi$. The dependence $\varphi_{ \approx } = f\left( {\left( {p_{r}^{ * } } \right)_{ \approx } } \right)$ also has an application, which is to determine the minimum threshold value $\left( {p_{r}^{ * } } \right)_{ \approx }$, at which the value of the criterion $\varphi$ will not be less than the specified value of $\varphi_{0}$. For the investigated information and communication system, this definition is embodied in the expression

30 $$ \left( {p_{r}^{ * } } \right) \ge {{\left( {\varphi - 0.3562} \right)} \mathord{\left/ {\vphantom {{\left( {\varphi - 0.3562} \right)} {\left( {\varphi_{0} - 0.3795} \right)}}} \right. \kern-\nulldelimiterspace} {\left( {\varphi_{0} - 0.3795} \right)}}. $$

Thus, as an experiment, we investigated the model of operation of the cyber-physical system of the Situation Center of DIT of VCC in the metrics of functional safety, formalized in “Models and methods”.

## Discussion

Let's start the discussion of the results presented in “Results” with a brief excursion into their theoretical background. Thus, we calculated the estimates of the metric $\left\{ {\tau ,\varphi } \right\}$ ($\tau$ is the mathematical expectation of the time till the cyber-physical system inoperation, *φ* is the mathematical expectation of risk factor that describes the losses from the probable fact of implementation of the failure despite the operation of protection mechanisms that cause inoperation) for the cyber-physical system of the Situation Center of DIT of VCC. It is characterized by the content of the conglomerate of sets $\{ Q,D,R,U\}$ and the value of the duration of the cycle $\Delta t$ (the minimum time interval after which the investigated system can change its state).

The availability of a statistically representative amount of information on the operation of the investigated system allowed experts to classify potential failures in the VPR metric. These circumstances allowed us to move from the direct calculation of the criteria of the metric $\left\{ {\tau ,\varphi } \right\}$ by expressions (21), (22) to the calculation of estimates of these criteria $\left\{ {\tau_{ \approx } ,\varphi_{ \approx } } \right\}$ by expressions (24), (25). To calculate the estimates $\{ \tau_{ \approx } ,\varphi_{ \approx } \}$ for the investigated system by expressions (23), the elements of the sets:—$\left\langle {T^{ * } } \right\rangle$ (mathematical expectations of the number of cycles between the respective failures), $\left\langle {D^{ * } } \right\rangle$ (mathematical expectations of the number of cycles required by protection mechanisms to neutralize the corresponding failures), $\left( {p_{r}^{ * } } \right)$ (share of successfully neutralized failures); were previously calculated for the investigated system by expressions (23).

The presented in Fig. 2 information shows that defined in the VPR-metrics of the risk assessment of the identified threats to the investigated system differ significantly in terms of the values of this general characteristic and the mechanisms for implementing the relevant failures. Direct analysis of this information without the mathematical apparatus presented in “Models and methods” does not allow to establish a functional relationship between the information in Fig. 2 and the values of the indicators of the functional safety attribute. Thus, the relevance of our research was reaffirmed.

The presented in Fig. 3 information shows that the increase in the values of both parameter $\left\langle {T^{ * } } \right\rangle_{ \approx }$ and parameter $\left( {p_{r}^{ * } } \right)_{ \approx }$ positively affect the value of the criterion $\tau_{ \approx }$, which characterizes the assessment of the mathematical expectation of the time till the investigated cyber-physical system inoperation. Obviously, the greater the parameter value $\left\langle {T^{ * } } \right\rangle_{ \approx }$, the greater the interval between failures, i.e., the intensity of the negative impact on the investigated system decreases. At the same time, the growth of the parameter $\left( {p_{r}^{ * } } \right)_{ \approx }$ indicates an increase in the share of successfully neutralized failures, i.e., positively characterizes the configuration scheme and architecture of the protection subsystem of the investigated system.

The condition (29) defined for the investigated system also positively affects the practical orientation of the criterion $\tau$. With its help, for example, it is easy to see that for the value of the criterion *τ* for the investigated system to be greater than $\tau_{0} = 200 \times 24$ [h], it is necessary that the inequality $\left( {p_{r}^{ * } } \right)_{ \approx } \ge 0.9879 - 0.005\left\langle {T^{ * } } \right\rangle_{ \approx }$ be satisfied, i.e., at $\left\langle {T^{ * } } \right\rangle_{ \approx } = 30 \times 24$ [h] we has $\left( {p_{r}^{ * } } \right)_{ \approx } \ge 0.8379$ and at $\left\langle {T^{ * } } \right\rangle_{ \approx } = 60 \times 24$ [h] we have $\left( {p_{r}^{ * } } \right)_{ \approx } \ge 0.6879$. But unfortunately, the parameter $\left( {p_{r}^{ * } } \right)_{ \approx }$ is a general qualitative characteristic of the protection subsystem. In this research, we do not give recommendations on how to organize this subsystem and do not assess whether the calculated value $\left( {p_{r}^{ * } } \right)_{ \approx }$ is achievable in principle.

The presented in Fig. 4 information shows that the risk factor acquires its maximum value $\varphi_{ \approx } = 0.3562$ at $\left( {p_{r}^{ * } } \right)_{ \approx } = 0$, i.e. if the protection subsystem functions perfectly or negative effects on the investigated system are completely absent (relatively close to reality example of such a situation is the operation of the investigated system isolated from the global network) then the risk factor acquires the minimum value of $\varphi_{ \approx } = 0.2$ for the investigated system at $\left( {p_{r}^{ * } } \right)_{ \approx } = 0.8702$.

The result proves the obvious fact that even an ideal protection subsystem is not a basis for claiming that the target system is guaranteed against inoperation. Thus, the antagonism of the “second law of thermodynamics vs. perpetuum mobile” also works for cyber-physical systems. Another advantage of Fig. 4 is clear—the more convex the curve $\varphi _{ \approx }  = f\left( {\left( {p_{r}^{ * } } \right)_{ \approx } } \right)$, the more efficient the protection subsystem.

So, we have described two functional security metrics based on the Markov model for the operation of a cyber-physical system. We have also shown that using the general VPR vulnerability system, the parameters of this model can be effectively estimated based on a small amount of empirical data, which is an undeniable advantage compared to, for example, the expert assessment method. Of course, the model we have considered has several assumptions related, in particular, to the impossibility of the simultaneous occurrence of several failures, as well as their independence from each other. Our further work will be aimed at weakening these assumptions and obtaining a more complex and generalized model, the dynamics of which will be as close as possible to the behaviour of real systems.

Finally, it should be noted that the technology of functional safety assessment based on the Markov model of cyber-physical system operation proposed in the article is based on generally accepted, valid, updated VPR-metrics and proved to be an adequate mathematical apparatus of Markov chains. These facts, and the rigor and reversibility of the analytical transformations made in the formalization of the metric $\left\{ {\tau ,\varphi } \right\}$ substantiate the adequacy of the mathematical apparatus presented in the article.

## Conclusions

The assessment of functional safety is one of the primary tasks both at the design stage and at the stage of operation of critical infrastructure at all levels. The article's main contribution is the information technology of calculating the author's metrics of functional safety for estimating the instance of the model of the cyber-physical system operation. The calculation of metric criteria (mathematical expectation of cyber-physical system operation to failure and risk factor) analytically summarizes the results of expert evaluation of the system in VPR-metrics and the results of statistical processing of information on the system's operation presented in the parametric space Markov model of this process. The advantage of the author's approach over analogues is:

- the need to process orders of magnitude less empirical data to obtain objective estimates of the investigated system;
- taking into account the configuration scheme and architecture of the security subsystem of the investigated system when calculating the metric;
- completeness, compactness, and simplicity of interpretation of evaluation results;
- the ability to assess the achievability of the limit values of the metric criteria based on the model of operation of the investigated system.

As an example, the article demonstrates the author's technology to assess the functional safety of the model of a real cyber-physical system of the Situation Center of the Department of Information Technology of Vinnytsia City Council (Vinnytsia, Ukraine).

However, in formalizing the Markov model of cyber-physical system operation, attackers believed that vulnerabilities used to lead to failures or inoperation were independent. The probable situation of simultaneous exploitation of one vulnerability by more than one attacker was also not considered. Considering these circumstances in the mathematical apparatus presented in the article is the direction of further research.

## Data Availability

The datasets for the analyzed during the current study are available in CVE Details: the ultimate security vulnerability data source repository: https://www.cvedetails.com/. All data on the link is in the public domain. In our study, we used data on such vulnerabilities as: CVE-2019-9511, CVE-2015-5206, CVE-2019-9512, CVE-2020-9481, CVE-2020-17509, CVE-2008-0127, CVE-2007-6593, CVE-2021-36301, CVE-2019-18805, CVE-2017-6745, CVE-2021-45253, CVE-2022-22055, CVE-2021-45814, CVE-2021-44599, CVE-2020-0060.
